# IS THERE A DIFFERENCE BETWEEN TERMINAL LUCIDITY AND PARADOXICAL LUCIDITY?

**DOI:** 10.1093/geroni/igac059.354

**Published:** 2022-12-20

**Authors:** Andrew Peterson, Justin Clapp, Kristin Harkins, Melanie Kleid, Emily Largent, Shana Stites, Jason Karlawish

**Affiliations:** George Mason University, Fairfax, Virginia, United States; University of Pennsylvania Perelman School of Medicine, Philadelphia, Pennsylvania, United States; University of Pennsylvania Perelman School of Medicine, Philadelphia, Pennsylvania, United States; University of Pennsylvania Perelman School of Medicine, Philadelphia, Pennsylvania, United States; University of Pennsylvania Perelman School of Medicine, Philadelphia, Pennsylvania, United States; University of Pennsylvania Perelman School of Medicine, Philadelphia, Pennsylvania, United States; University of Pennsylvania Perelman School of Medicine, Philadelphia, Pennsylvania, United States

## Abstract

Recent arguments in aging and dementia research suggest that “terminal lucidity”—defined as unexpected communication or connectedness occurring shortly before death—is distinct from “paradoxical lucidity”—defined as an episode of communication or connectedness in a person who is assumed to have lost these capacities due to progressive neurodegeneration. We disagree with this distinction and argue that terminal lucidity is a special subtype of paradoxical lucidity. We suggest that specifying the relationship between terminal and paradoxical lucidity is important for investigating the underlying mechanism of lucidity in dementia.

